# Re-Examination of Chinese Semantic Processing and Syntactic Processing: Evidence from Conventional ERPs and Reconstructed ERPs by Residue Iteration Decomposition (RIDE)

**DOI:** 10.1371/journal.pone.0117324

**Published:** 2015-01-23

**Authors:** Fang Wang, Guang Ouyang, Changsong Zhou, Suiping Wang

**Affiliations:** 1 Center for Studies of Psychological Application and School of Psychology, South China Normal University, Guangzhou, China; 2 Key Laboratory of Mental Health and Cognitive Science of Guangdong Province, South China Normal University, Guangzhou, China; 3 Department of Physics, Hong Kong Baptist University, Kowloon Tong, Hong Kong, China; 4 Centre for Nonlinear Studies and the Beijing–Hong Kong–Singapore Joint Centre for Nonlinear and Complex Systems (Hong Kong), Institute of Computational and Theoretical Studies, Hong Kong Baptist University, Kowloon Tong, Hong Kong, China; University of Leicester, UNITED KINGDOM

## Abstract

A number of studies have explored the time course of Chinese semantic and syntactic processing. However, whether syntactic processing occurs earlier than semantics during Chinese sentence reading is still under debate. To further explore this issue, an event-related potentials (ERPs) experiment was conducted on 21 native Chinese speakers who read individually-presented Chinese simple sentences (NP1+VP+NP2) word-by-word for comprehension and made semantic plausibility judgments. The transitivity of the verbs was manipulated to form three types of stimuli: congruent sentences (CON), sentences with a semantically violated NP2 following a transitive verb (semantic violation, SEM), and sentences with a semantically violated NP2 following an intransitive verb (combined semantic and syntactic violation, SEM+SYN). The ERPs evoked from the target NP2 were analyzed by using the Residue Iteration Decomposition (RIDE) method to reconstruct the ERP waveform blurred by trial-to-trial variability, as well as by using the conventional ERP method based on stimulus-locked averaging. The conventional ERP analysis showed that, compared with the critical words in CON, those in SEM and SEM+SYN elicited an N400–P600 biphasic pattern. The N400 effects in both violation conditions were of similar size and distribution, but the P600 in SEM+SYN was bigger than that in SEM. Compared with the conventional ERP analysis, RIDE analysis revealed a larger N400 effect and an earlier P600 effect (in the time window of 500–800 ms instead of 570–810ms). Overall, the combination of conventional ERP analysis and the RIDE method for compensating for trial-to-trial variability confirmed the non-significant difference between SEM and SEM+SYN in the earlier N400 time window. Converging with previous findings on other Chinese structures, the current study provides further precise evidence that syntactic processing in Chinese does not occur earlier than semantic processing.

## Introduction

Language comprehension involves not only single word recognition but also semantic integration of words according to certain syntactic rules. One of the core concerns in human sentence comprehension is the relative time course of and interplay between semantic and syntactic processing [1–6]. A large number of studies have been carried out on Indo-European languages, however, there is still much debate regarding the interplay between semantic and syntactic processes. Some studies have suggested that they are relatively independent, with different eye movement patterns and distinct brain systems [7–11], while others found that failed syntactic category processing appears to block lexical-semantic integration, suggesting the primacy of syntax over semantics [12–16]. In contrast, with respect to the issue of the relative time course, the results of studies from alphabetic languages seemed to support the view that both semantic and syntactic processes occur relatively fast [17], and differ from each other in the time course, with syntactic processing initiates earlier than the semantics [10, 12, 18]. Consider an English auditory ERP experiment reported in Hahne et al. [12], where participants listened to sentences which were either correct, semantically incorrect, syntactically incorrect, or both semantically and syntactically incorrect. Results showed that, independent of semantic constraints and task requires, syntactic processing could be initiated very early.

It is worth noting that, unlike Indo-European languages, Chinese is an isolating language, and therefore has very little explicit morphology (e.g. no case marking or inflectional indicators, and no intra-sentence concordance rules) [19]. Apart from this, Chinese permits a number of word order permutations. Given the unique properties of this logographic writing system, in recent years Chinese, especially Chinese syntactic processing, has attracted many psychologists’ attention [5, 6, 20–22]. Some early studies on Chinese syntactic processing tried to adopt exactly the same logic used in Indo-European studies in manipulating a pure syntactic violation condition [14, 23]. However, the lack of explicit grammatical markers inevitably leads to a result in which any syntactic violation in Chinese is accompanied by a change of meaning. Thus, some researchers have suggested that using a double violation paradigm would be a better choice to study Chinese syntactic processing [6, 19, 21]. Specifically, this double violation paradigm includes congruent control (CON), semantic violation (SEM), and double violation (SEM+SYN) conditions. Importantly, in studies using this paradigm the semantic disruption degree was carefully matched in the two violation conditions (i.e. SEM and SEM+SYN). Thus, any difference observed between SEM and SEM+SYN can be interpreted as a syntactic effect. Employing this modified double violation paradigm, most studies seemed to support the view that, at least for Chinese, syntactic processing exerts influence until the relative late time window [20, 21]. For example, Yang et al. [21] found that the difference between SEM and SEM+SYN could only be shown in eye movement patterns in the post target region. However, there was an important confound in these studies. Specifically, the researchers used different grammatical categories of critical words when comparing the SEM and SEM+SYN conditions; for example, a noun might be presented in SEM and a verb in SEM+SYN [20, 21]. Given the documented processing difference between different word categories [24, 25], the conclusions from these studies are less convincing. To overcome this confounding issue, some researchers applied same syntactic categories of critical words across different experimental conditions. For example, to investigate whether syntactic sub-categorization (transitivity) necessarily proceeds semantic processing, Zhang et al. (2013) created three conditions: Correct (CORRECT), Semantic only anomaly (SEMANTIC), and Transitivity plus semantic anomaly (TRANSITIVITY). The transitive verb in the correct sentence was replaced by a transitive but semantically anomalous verb, or by an intransitive verb, creating SEMANTIC and TRANSITIVITY, respectively. They found a broad negativity in the 300–500 ms range (N400) for the SEMANTIC and TRANSITIVITY condition [26]. In this study, however, the semantic violation in the two violation conditions (i.e., SEMANTIC and TRANSITIVITY) were not carefully matched to the same degree. Hence, they could only suggest semantic integration proceeds when the processing of transitivity fails rather than effectively infer the precise relative timing of the syntactic processing in Chinese comprehension.

Recently, using the same double violation logic, Wang et al. [6] further studied Chinese syntactic processing in two Chinese verb-argument structures (i.e. the NP1-ba/bei-NP2-VP construction). Similarly, they created a SEM by using a semantically violated transitive verb after the NP1-ba/bei-NP2 frame, and created a SEM+SYN by using an intransitive verb after the same frame. But they also carefully matched the semantic disruptions in the two violation conditions (i.e. SEM and SEM+SYN) to the same degree. The results showed a similar N400 for SEM and SEM+SYN, suggesting that introducing the SYN did not interrupt semantic processing. However, it is possible that the verb sub-categorization violation in the SEM+SYN condition could be casted either as a syntactic category violation or a semantic violation, or both [15, 27–30]. In Wang et al. (2013) study [6], the nature of the violation hinges upon the precise form of the syntactic prediction made at the NP2 preceding the critical verb. In the co-verb structure tested in Wang et al. (2013), given the partial input “NP1-ba/bei-NP2”, if the parser made a very specific syntactic prediction about the transitivity of the upcoming verb (i.e. it should be a transitive verb), then syntactic violation would occur once an intransitive verb was introduced. On the other hand, if the syntactic expectation at the NP2 was only about an upcoming verb but without fully specifying the transitivity information, both syntactic and semantic integration difficulty could arise when an intransitive verb was integrated into the current sentence. The fact is that the syntactic problem of missing arguments would naturally lead to the semantic integration problem that no coherent meaning could be derived. The design and findings in Wang et al. (2013) were unable to tease these possibilities apart. Therefore, other stricter manipulations of syntactic violation should be considered in this line of research.

In the present study, we built on the Wang et al. [6] study by further examining the nature of semantic and syntactic processing in Chinese using a methodology that eliminates an important confound in earlier research. We adopted the modified double violation paradigm and manipulated the verb transitivity in a verb-argument structure, but we used the canonical “NP1+VP+NP2” structure. In this structure, although similarly manipulated the transitivity of verbs, the NP2 rather than VP served as the critical words. That is, the (in)transitive verb was already processed before the critical NP2. Therefore the readers could make a relatively clear prediction about whether there would be an upcoming NP2 no matter through semantic or syntactic analysis. Specifically, when the verb is transitive, a subsequent NP2 is expected, whereas no such prediction would be made if the verb is intransitive (because an NP2 in this case might create a syntactic violation). Further, unlike Wang et al. [4] and Yang et al. [21], the target word was always a noun (NP2), which could avoid the confounding effect introduced by comparing different syntactic categories of critical words in different conditions [25, 27].

Another novel aspect of the current study is that a new method, Residue Iteration Decomposition (RIDE) [33–36], was applied to the present data in addition to the conventional ERP analysis. The motivation to use RIDE is that several previous studies using the conventional ERP analysis reported similar effects in the N400 time window when comparing SEM with SEM+SYN, which might be the result of the smearing effect caused by trial-to-trial variability; that is, the trial-to-trial latency variability of ERP components could diminish the ERP amplitude after averaging. As a result, the cross-conditional difference may also be attenuated [33]. Compared with the conventional ERP analysis, RIDE was developed to detect and retrieve the latency-variable components and then reconstruct the ERP after re-synchronizing each component to the most probable latency, therefore compensating for the trial-to-trial latency variability. After RIDE analysis, the smearing effect due to the trial-to-trial latency variability is greatly reduced and the cross-conditional difference reflects purer amplitude difference. Therefore, the present study took advantage of the RIDE method in combination with conventional ERP analysis, to further investigate the effects elicited by SEM and SEM+SYN conditions in N400 and P600 time windows.

In sum, in this study, we are particularly interested in two questions: (1) Does syntactic processing in Chinese occur earlier than semantic processing, and (2) Could the effects elicited by SEM and SEM+SYN be influenced by trial-to-trial latency variability? With respect to the first question, we manipulated the transitivity of verbs in Chinese simple sentences (NP1+VP+NP2) to introduce semantic and syntactic violation. Based on previous studies [15, 31–32, 37, 41], LAN, N400 and P600 are the three candidate components that are potentially related to the syntactic effect (i.e., the difference between SEM and SEM+SYN). LAN is an early component that is traditionally associated with morphological or syntactic processing; N400 is traditionally associated with semantic anomaly but has more recently tied to syntactic processing as well; and P600 is a late component traditionally associated with syntactic anomaly. If syntactic structure-building precedes semantic processing, we should be able to find some differences between the two types of violation in a relatively early time window. Specifically, compared with the congruent condition, the SEM+SYN condition might elicit an early component (e.g., LAN) while SEM would elicit an N400; or both conditions evoke an N400 with different amplitudes or scalp distributions. However, if the embedded syntactic violation cannot be detected immediately, differences between SEM+SYN and SEM might be observed in a relatively late time window. In that case, both SEM and SEM+SYN may elicit a similar N400, but a P600 might only be observed in SEM+SYN; or both violation conditions evoke an N400-P600 pattern, with different amplitudes of P600 in SEM+SYN. As for the question of potential trial-to-trial latency variability influence, we used both traditional ERP analysis and the newer RIDE method to further and precisely investigate the effects elicited by SEM and SEM+SYN conditions.

## Materials and Methods

### Ethics Statement

This study was approved by the Psychology Research Ethics Committee of South China Normal University. The participants provided written informed consent prior to the experiment.

### Participants

Twenty-one right-handed students (4 males, mean age = 21 years) from South China Normal University were paid to participate in this experiment with informed consent. All were native speakers of Mandarin Chinese, had no reading disabilities, and had normal or corrected-to-normal vision.

### Materials

The critical materials consisted of one hundred and twenty sets of sentences. Each sentence frame was used to create three types of sentences: congruent sentences (the CON condition), sentences with a semantic violation (the SEM condition), and sentences with combined semantic and syntactic violations (the SEM+SYN condition) (see Table 1 for examples). These sentences were divided into three lists, each containing 40 sentences for each of the three experimental conditions. Within each list, each sentence stem was presented only once, and across the three lists, each sentence stem appeared in all three conditions. To counterbalance the number of congruent and violated sentences, an additional 40 congruent filler sentences (with similar structures as the experimental sentences) were added. Consequently, each list included 160 sentences in total. The order of the sentences was randomized once for each list and then presented in the same order to participants.

**Table 1:** Experimental conditions and example sentences.

Condition	Sentence
CON	警方*揭穿* **骗局**之后人群就散去了。
	After police *debunked* the **fraud** the crowd dispersed.
SEM	警方*掀起* **骗局**之后人群就散去了
	After police *raised* the **fraud** the crowd dispersed.
SEM+SYN	警方*交战* **骗局**之后人群就散去了
	After police *fought* the **fraud** the crowd dispersed.

The example sentences are in Chinese, with literal English translation in brackets. The critical words are in bold. The verbs are in italic. CON, congruent condition; SEM, semantic violation condition; SEM+SYN, combined semantic and syntactic violation condition.

All the critical verbs selected were those that could be used as either transitive or intransitive. As shown in Table 2, critical verbs in the three conditions were well matched across the three conditions in word frequency, stroke number and concreteness (all with *F*s <1).

**Table 2:** Mean word frequency (WF, in units of occurrence per million), mean number of strokes (NS), and mean concreteness (CO) of the critical verbs for the three conditions.

Condition	WF	NS	CO
CON	9.95(18.22)	8.82(2.37)	3.34(.71)
SEM	10.13(18.80)	8.60(2.27)	3.31(.75)
SEM+SYN	10.14(18.48)	8.50(2.27)	3.42(.83)

The standard deviations are shown in parentheses. CON, congruent condition; SEM, semantic violation condition; SEM+SYN, combined semantic and syntactic violation condition.

### Rating

To check the validity of the critical materials, we conducted three rating studies to determine (1) the semantic plausibility of the complete sentences across the three experimental conditions, (2) the plausibility of subject—predicate construction, and (3) the difficulty of continuing the sentences after the critical verb.

It was important to ensure that the semantic plausibility of the complete sentences was comparable in the two violation conditions because some of the violation effect may be delayed until the end of the sentence, and local semantic violation may result in different degrees of global incongruence. A different group of 24 participants was asked to rate the semantic plausibility of the complete sentences on a 5-point scale (ranging from 1 = *extremely unacceptable* to 5 = *fully acceptable*). Table 3 shows the results. Repeated measures ANOVA revealed a significant main effect of condition [*F*(2, 357) = 1674.833, *p* <.001]. Pair-wise comparisons indicated that congruent sentences were rated as more acceptable than the other two conditions (*p*s <.001) whereas the two violation conditions received similar mean scores, suggesting that the degree of semantic violation was matched between these two conditions.

**Table 3:** Mean scores and standard deviations in the three rating: the plausibility of complete sentences (Rating 1) for the three conditions, the plausibility of subject—predicate construction (Rating 2), and the difficulty to continue the sentences after the critical verb (Rating 3).

Condition	Rating 1	Rating 2	Rating 3
CON	4.43(.36)	4.18(.39)	4.45(.40)
SEM	1.79(.40)	4.16(.37)	2.37(.64)
SEM+SYN	1.81(.46)	4.21(.51)	2.29(.64)

The standard deviations are shown in parentheses. CON, congruent condition; SEM, semantic violation condition; SEM+SYN, combined semantic and syntactic violation condition. Ratings 1 and 2 ranged from 1 = ‘‘extremely unacceptable” to 5 = ‘‘fully acceptable”; Rating 3 ranged from 1 = ‘‘very difficult” to 5 = ‘‘very easy”.

To make sure violation would not occur until the critical words were presented in each of the three experimental conditions, a separate group of 24 participants was asked to rate the plausibility of the subject-predicate construction of the sentences. That is, we presented participants sentence frames up to (but not including) the critical word and asked them to rate plausibility on a 5-point scale, with 1 = *completely unreasonable* and 5 = *quite reasonable*. The results are shown in Table 3. Analysis of variance (ANOVA) showed that there was no significant main effect of condition [*F*(2, 357) = 5.425, *p* >.5]. In addition, the plausibility rating of all subject-predicate constructions was above 3 on the 5-point scale, suggesting that the subject-predicate constructions we used in our materials were all plausible.

Given that all the critical words were at the middle of the experimental sentences, there was a possibility that sentences with local semantic incongruence would turn out to be globally congruent. Therefore we conducted another rating to make sure that (1) both types of semantic violation could be detected at the point the critical verbs appeared and (2) in both violation conditions, readers had a similar expectancy about whether the information after critical words could eliminate the local anomaly. Another group of 24 participants was recruited to rate the first part of the sentences up to (and including) the critical word on a 5-point scale (ranging from 1 = *very difficult* to 5 = *very easy*) according to ‘‘how difficult it is to continue the sentence as a congruent one.”. The results are shown in Table 3. Repeated measures ANOVA revealed a significant main effect of condition [*F*(2, 357) = 554.797, *p* <.001]. Pair-wise comparisons indicated that whereas SEM and SEM+SYN were rated as more difficult to continue than CON (*p*s <.001), the first two had similar mean scores, suggesting that the degree of severity of violation at the critical words was well-matched between SEM and SEM+SYN, and readers had a similar expectancy that the information after critical words could not eliminate the local anomaly.

### Procedure

Participants were randomly assigned to one of three stimulus lists and were tested individually in a sound-attenuating, electrically shielded booth. Before the experiment started, a brief practice session of 20 sentences was used to familiarize participants with the procedure and the task. Participants were instructed to minimize eye and body movements throughout the experiment, and to restrict them to the break periods. Sentences were presented word by word at the center of the screen. Each trial began with the presentation of a fixation cross at the center of the screen for about 200 ms, followed by a 200 ms blank screen, and then followed by the first constituent. Each word appeared on the screen for 400 ms with an inter-stimuli interval (ISI) of 300ms. The sentence’s final target word was presented by itself. After a blank-screen of 500 ms following the period, participants were then cued by a question “Is this sentence reasonable?” to decide whether the sentence was plausible or not by pressing the “YES” or the ‘‘NO” button on a response box. The assignment of plausible and implausible response to the left and right hand was counterbalanced. Participates pressed the “SPACE” key to begin the next trial. The whole experiment lasted about 80 minutes.

### Electroencephalography (EEG) recording

EEG was recorded from the following 38 sites according to the international 10–20 system: FP1, FPz, FP2, AF7, AF3, AF4, AF8, F7, F3, Fz, F4, F8, FT7, FC3, FCz, FC4, FT8, T7, C3, Cz, C4, T8, TP7, CP3, CPz, CP4, TP8, P7, P3, Pz, P4, P8, PO7, PO5, POz, PO6, PO8, and Oz. EEG response was referenced to the left mastoid online, but re-referenced offline to the average of the two mastoids. The electro-oculogram (EOG) was obtained from below vs. above the left eye (vertical EOG). The AFz electrode on the cap served as ground. Electrode impedances were always kept below 5 KΩ. The EEG and EOG signals were digitized online with a sampling frequency of 500 Hz and filtered digitally with a 0.02 to 30 Hz band pass offline. Epochs with amplitudes exceeding ±80μV were excluded from the averages through artifact rejection.

### Data analysis with conventional averaging ERPs

For data analysis, the epoch was 1200 ms, ranging from 200 ms before the onset of the critical words to 1000 ms after them. Two time windows were chosen for data analysis with conventional averaging ERP based on visual inspection and previous studies for the possible ERP effects: 300–500 ms for possible N400 and LAN effects, and 570–810 ms for possible P600 effects.

For each time window, repeated-measures ANOVAs were carried out separately for midline and lateral electrode sites. The ANOVA for midline electrodes was performed with two factors: condition (CON, SEM, and SEM+SYN) and electrode site (8 levels: FPz, Fz, FCz, Cz, CPz, Pz, POz, and Oz). The ANOVA for lateral electrodes included three factors: condition (CON, SEM, and SEM+SYN), region (anterior, central, and posterior), and hemisphere (left and right). Crossing the factors of hemisphere and region produced six regions of interest (ROI), each containing four lateral electrodes: left anterior (F7, F3, FT7, FC3), right anterior (F4, F8, FC4, FT8), left central (T7, C3, TP7, CP3), right central (C4, T8, CP4, TP8), left posterior (P7, P3, PO7, PO3), and right posterior (P4, P8, PO4, PO8). Data were averaged within each ROI for each participant before statistical analysis. Comparisons were planned for each ROI if interactions reached significance. The Greenhouse-Geisser correction was applied when evaluating effects with more than one degree of freedom in the numerator.

### ERP processing with RIDE

In the present paper we employed the algorithm of RIDE from Ouyang et al. [33]. The toolbox of the latest RIDE algorithms is available at http://cns.hkbu.edu.hk/RIDE.htm. The specific algorithms and settings of the RIDE processing are as follows: we separated ERP into three component clusters: S for capturing the stimulus-locked component cluster, C1 component cluster for capturing the N400 complex and C2 component cluster for capturing the P600 complex. The latency of S for each single trial was set to be locked to the stimulus onset itself. The latency of components C1 and C2 for single trials was firstly estimated by Woody’s method within the time windows [200 ms, 600 ms], and [400 ms, 800 ms], respectively. After the latencies for the three component clusters were obtained, the data were subject to RIDE decomposition [33] into three component clusters associated with the three latency sets. Then the latencies of C1 and C2 for single trials were updated by the cross-correlation between the template of C1 and C2 and single trial ERP after removal of other components (also refer to [33]). The decomposition step and latency updating step were iterated until convergence.

## Results

### Behavioral results

Three participants were removed from all data analyses due to low accuracy (below 85%) on the semantic plausibility judgment task. Among the 18 participants left, two repeated measures ANOVAs showed that the main effect of condition was not significant for either the mean reaction time or the accuracy of the judgment task (*F*s <1, Table 4). Behavioral results suggested that participants closely attended to the stimuli.

**Table 4:** Mean reaction time (in milliseconds) and accuracy (percentages of correct) for the semantic plausibility judgment task in the three conditions.

Condition	Reaction time (ms)	Accuracy (%)
CON	456(329)	95.3(21.2)
SEM	436(268)	95.3(21.2)
SEM+SYN	447(288)	95.6(20.6)

The standard deviations are shown in parentheses. CON, congruent condition; SEM, semantic violation condition; SEM+SYN, combined semantic and syntactic violation condition.

### ERP data

**Fig 1:**
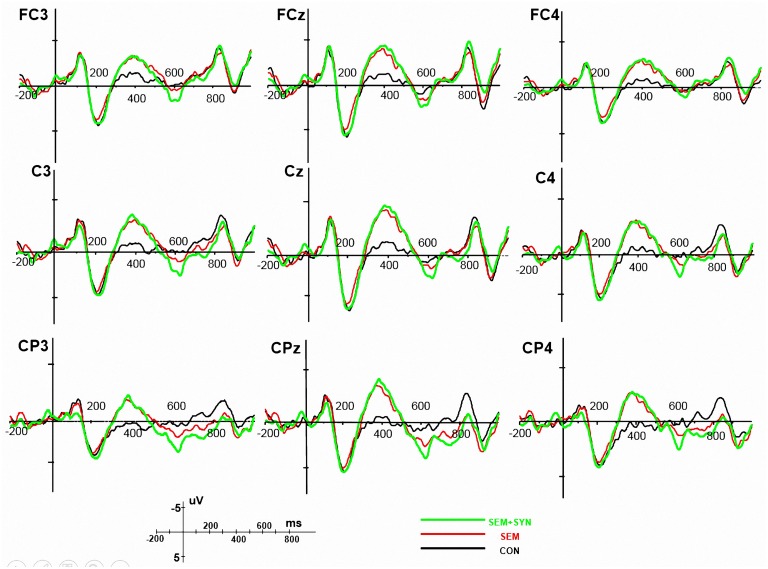
Grand average of ERPs. Average ERPs from the onset of the critical words (NP2) up to 1000 ms thereafter for congruent condition (CON, black line), semantic violation condition (SEM, red line), and combined semantic and syntactic violation condition (SEM+SYN, green line) at 9 representative electrodes. Negativity is plotted upwards.

**Fig 2:**
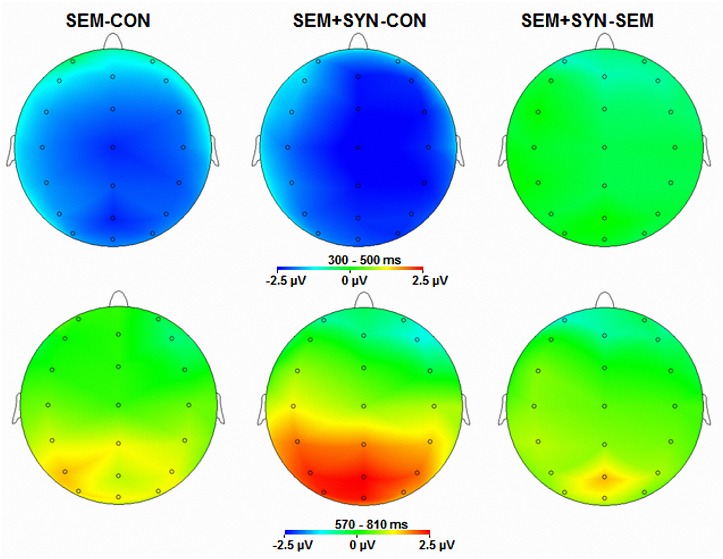
Topographic distributions of the mean ERP differences from 300 to 500 ms and those from 570 to 810 ms, respectively. “SEM-CON” = difference between SEM and CON, ‘‘SEM+SYN-CON” = difference between SEM+SYN and CON, and ‘‘SEM+SYN-SEM” = difference between SEM+SYN and SEM.

Figs. 1 and 2 show grand-average ERPs and scalp distribution in the CON, SEM, and SEM+SYN conditions in the 300–500 ms time window and the 570–810 ms time window. Compared with CON, both violation conditions showed a larger negative-going component peaking at around 400 ms (N400) with a broad bilateral distribution. In both violation conditions, the negativity was followed by a large positive-going wave starting from approximately 500 ms, which was largest over centro-parietal sites (P600). The amplitude of the P600 in SEM+SYN appeared to be larger than that in SEM.

### Time window 300–500 ms

The global ANOVA for midline electrodes showed a significant main effect of condition [*F*(2, 34) = 14.300, *p* <.001, η^2^ = .457] and a significant condition × electrode interaction [*F*(14, 238) = 2.341, *p* <.01, η^2^ = .121]. Separate ANOVAs at each electrode showed that the main effect of condition was significant at all midline electrodes (except for FPz, all *p*s <.01). Planned comparisons carried out at the seven electrodes with a significant main effect showed that, relative to the critical words in CON, those in SEM and SEM+SYN evoked a pronounced negativity (all *p*s <.05), but no significant difference was found between the two violation conditions (all *p*s >.2).

The global ANOVA for lateral electrodes showed a significant main effect of condition [*F*(2, 34) = 17.190, *p* <.001, η^2^ = .503]. Further simple main effect tests on condition showed that, in each region, the N400 area amplitude in the SEM and SEM+SYN condition was larger than that in the CON condition (left anterior region: *F*(2, 34) = 11.051, *p* <.001, η^2^ = .394; right anterior region: *F*(2, 34) = 10.459, *p* <.001, η^2^ = .381; left posterior region: *F*(2, 34) = 7.138, *p* <.01, η^2^ = .296; right posterior region: *F*(2, 34) = 13.250, *p* <.001, η^2^ = .438; left central region: *F*(2, 34) = 12.693, *p* <.001, η^2^ = .427; right central region: *F*(2, 34) = 16.223, *p* <.001, η^2^ = .488), and no significant difference was found between the two violation conditions (all *p*s >.1). These findings suggested that although the syntactic processing in SEM+SYN condition was failed, the semantic processing could nevertheless take place without additional influence.

### Time window 570–810ms

The global ANOVA for midline electrodes showed a significant main effect of condition [*F*(2, 34) = 4.591, *p* <.05, η^2^ = .213] and a significant condition × electrode interaction [*F*(14, 238) = 4.125, *p* <.005, η^2^ = .195]. Separate ANOVAs at each electrode showed that the main effect of condition was significant at four midline electrodes, namely CPZ, PZ, POZ, OZ [all *F*s >3.995, *p*s <.05]. Planned comparisons carried out at the 4 electrodes with a significant main effect showed that, relative to the critical words in CON, those in SEM and SEM+SYN evoked a pronounced positivity (except for PZ, all *p*s <.05). The difference between SEM and SEM+SYN was significant at PZ and POZ electrodes (all *p*s <.05), with a larger positivity in SEM+SYN than in SEM.

The global ANOVA for lateral electrodes showed significant main effects of condition [*F*(2, 34) = 6.774, *p* <.01, η^2^ = .285] and region [*F*(2, 34) = 27.13, *p* <.001, η^2^ = .615], and a significant region × condition interaction [*F*(4, 68) = 9.763, *p* <.005, η^2^ = .365]. Further simple main effect tests on condition showed that, in the centro-parietal region, the P600 area amplitude in the SEM and the SEM+SYN conditions was larger than that in the CON condition at central and posterior regions [all *F*s >6.634, *p*s <.01], and the P600 area amplitude in the SEM+SYN condition was larger than that in the SEM condition at left posterior region [*F* (1, 17) = 4.449, *p* <.05, η^2^ = .209]. These results suggested that the additional syntactic violation in SEM+SYN did not exert influence until the later P600 time window.

### RIDE data

**Fig 3:**
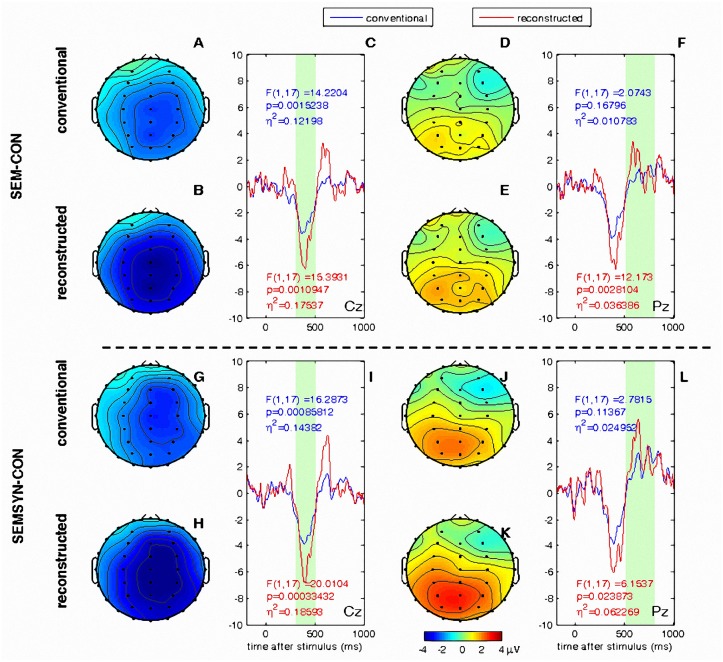
Statistical testing on conventional ERP and reconstructed ERP. A, B: The topography of the difference waves of conventional ERP and reconstructed ERP between SEM and CON averaged from the time window of [300ms, 500ms] (the green bar in C). C: The time courses of the difference waves of conventional ERP and reconstructed ERP between SEM and CON, from channel Cz. D-F: same results for the time window of P600 [500ms, 800 ms]. The time courses were from Pz. G-K: same results for the difference wave between SEM+SYN and CON. The digits are the F values, p values and η^2^ from the ANOVA test on the mean value averaged from the green bars. The blue digits are on conventional ERP and the red digits are on reconstructed ERP.

After processing of original ERP by RIDE, each ERP sub-component cluster defined by RIDE was re-synchronized to its own latency across single trials and was located at the most probable latency. Therefore the ERP was reconstructed by compensating for the trial-to-trial latency variability, and the new ERP was then called the reconstructed ERP. We then compared results based on the conventional ERP and the RIDE-reconstructed ERP. Fig. 3 shows the results of the global ANOVA comparing the control (CON) and violated conditions (SEM and SEM+SYN) for midline electrodes based on the two types of ERP, providing a direct comparison between conventional ERP and reconstructed ERP.

The topography differences between the control condition and violated conditions are shown for the conventional ERP (up) and reconstructed ERP (down), respectively (see Fig. 3). The topographies were averaged from a range of time windows based on the occurrence time window of N400 and P600 (the time windows being indicated by the light green bar behind the time courses). It can be clearly seen that the topography differences were darker in the reconstructed ERP produced by RIDE. The time courses show the ERP difference waves from a single electrode (indicated on the bottom right corner). Clearly, a higher amplitude and clearer biphasic pattern of N400 and P600 components are shown in the reconstructed ERP (red) than in the conventional ERP (blue).

In the time window of N400, pattern of results using the reconstructed ERP was generally consistent with results based on the conventional ERP, but there was a clearly enhanced statistical effect; in the time window of P600 between 500 ms and 800 ms, the changes as measured by the reconstructed ERP were dramatic. When the statistical effect was not significant in conventional ERP analysis (SEM-CON), the reconstruction method revealed an effect exceeding the boundary level. When the effect based on conventional analyses showed only a tendency toward significance (SEM+SYN-CON), the reconstruction revealed a strongly significant effect.

Neither conventional ERP nor reconstructed ERP showed a significant difference between the two violated conditions in the N400 time window. While in the P600 time window, the reconstructed ERP confirmed the amplitude effect between the two violation conditions between 570 and 810 ms. This finding is consistent with the assumption that the additional syntactic violation in SEM+SYN did not exert influence in the N400 or earlier time window.

Compare with the conventional average analysis, RIDE revealed a stronger effect of the already significant N400 effect and moved the time window of the significant P600 effect (between violation and control conditions) from 570–810ms to 500–800ms. Overall, the lack of a difference between SEM and SEM+SYN in the earlier N400 time window suggests that syntactic processing in Chinese does not necessarily occur earlier than semantic processing.

## Discussion

The majority of previous studies examining Chinese syntactic and semantic processing have used NP1-ba/bei-NP2-VP stimuli, as they have a fixed structure and place a relatively high constraint on the transitivity of the verb in the VP position [3, 6,14,23]. There have been a few eye movement [21] and fMRI [4] studies that have used the canonical SVO (NP1-VP-NP2) structure, creating the critical SEM+SYN violation condition by changing the object noun to a verb. However, these studies may have introduced a number of potential confounds, such as using critical words of different syntactic categories across different experimental conditions, and lists of words that vary in the number of verb arguments. (It is still unclear whether the latter case should be considered a semantic or syntactic violation.) In order to avoid these problems, the current study made three improvements to the methodology commonly used to study Chinese syntactic and semantic processing. First, the previous *ba/bei* structure was changed to the SVO construction and the transitivity of verbs was manipulated so that readers could make a relatively clear prediction about whether there would be an NP2 following the presented verb. This allows for the examination of Chinese syntactic processing through violation of syntactic predictions in a canonical SVO structure. Second, compared with the previous studies using the SVO structure, our study went a step further by keeping the critical words identical across three conditions, avoiding the confounds introduced in previous methods. Moreover, a new method-RIDE was employed to reconstruct ERP in un-smeared form so that we could provide clearer picture about the cross-condition amplitude effects.

Through the above manipulations and traditional and reconstructed ERP analyses, the following results were obtained. First, in the 300–500 ms time window, the two violation conditions, compared with the congruent condition, elicited an N400 of similar amplitude and distribution. Using the more sophisticated and sensitive RIDE analysis that compensated for the single-trial latency variability of the N400 component, our results confirmed that there was no difference between SEM and SEM+SYN within the N400 time window, suggesting that the underlying processes were indeed highly similar in these two violation conditions during this time window. With respect to the functional neural mechanism underlying the N400, it may reflect the difficulty of semantically integrating a word into its preceding context, consistent with numerous previous studies on words, sound, and picture processing [37,39,40]. Combined with the results of a pretest in which the degree of semantic violation was matched between the two violation conditions, these results demonstrate that the N400 effect observed in our experiment mainly reflects semantic processing; the introduced syntactic violation in SEM+SYN did not evoke additional effects in the early time window (i.e. the N400 or earlier). Thus, unlike the primacy of syntactic processing as shown in previous studies on Indo-European languages, Chinese semantic processing does proceed even when meeting failed syntax.

Second, within the 570–810 ms time window, the two violation conditions both elicited P600 effects widely distributed across the centro-parietal region; however, the P600 in SEM+SYN was larger than that in SEM. Furthermore, during the 500–800ms time window, RIDE revealed a stronger and earlier P600 effect in the violated condition relative to the congruent condition. Our findings seem to suggest that the effect of an additional syntactic violation does not occur until a later time window. The larger P600 effect for the SEM+SYN condition observed in the present study has at least two possible explanations. First, as sentences are processed, semantic and syntactic information interact and, when both semantic and syntactic violations exist, parsers may have difficulty integrating information into a final representation [15]. Second, the larger P600 effect may also reflect pure syntactic processing [31,41,42]. No matter which type of processing the larger P600 effect reflects, however, the present study suggests that the manipulation of syntactic violation did induce a separate type of processing, different from pure semantic processing. This additional P600 effect is consistent with other recent Chinese ERP studies (though these studies still have some confounding factors) [6,23]. To summarize, although syntactic information in Chinese is flexible and implicit, with vague grammatical properties and no explicit morphological inflections, syntactic processing is not equal to semantic processing and does not exert influence until a much later time window (i.e. the P600 time window), consistent with the results found in Indo-European languages [2,43].

Like Zhang et al. [23] and Wang et al. [6], we also observed an unexpected P600 effect for the semantic violation condition. Traditionally, it has been proposed that the P600 effect reflects syntactic reanalysis and repair [31,38], or processes of syntactic integration [44], but several recent studies have found that a pure semantic violation may also elicit a P600, with the caveat that this semantic P600 has always been reported for animacy violation or thematic role reversals [43,45–47]. In our experimental stimuli, despite strict control of these potential violations, there was still a P600 effect in the SEM violation. Thus, we suggest that the semantic P600 in the present study may reflect a domain-general, late-phase cognitive process, wherein the brain monitors, reanalyzes, and repairs any processing errors [48]. Specifically, when a parser encountered an unexpected argument mismatched with the verb in SVO structure, the conflict between two semantic representations (i.e., the predicted representation and the real one) may have elicited monitoring and resolution, reflected by the P600 effect. Another possibility is that the P600 may be evoked by the explicit plausibility judgment task [2].

Last but not least, as described earlier, apart from the traditional ERP analysis, we also used the more sophisticated and sensitive RIDE analysis, which has been successfully applied to experiments using priming [33], Go/Nogo [34], Simon task [35] and Oddball paradigms [36], to explore more deeply the ERP effects in language processing. By synchronizing each component to its most probable latency across single trials and averaging them, the RIDE analysis allows us to examine latency-corrected ERPs, so that we can compare the effect sizes much more meticulously. In our experiment, we applied this method to ERP data recorded using the RSVP paradigm. As it turned out, RIDE did show more sensitivity to effects by compensating for trial-to-trial latency variability, and it showed that the P600 effects elicited by semantic and syntactic violation occurred earlier than those demonstrated by the conventional analysis. But note that the effects evoked by SEM and SEM+SYN in the N400 time window were still similar, even after the correction for trial-to-trial latency variability, providing solid support for the previous results and conclusions acquired through traditional ERP analysis.

In conclusion, using a design that was not affected by confounds in earlier studies, as well as combining both the new method RIDE and the conventional averaging method to analyze the EEG data, the current results provide relatively strong evidence that the syntactic aspect of verb sub-categorization information was processed no earlier than semantics.

## References

[1] Hagoort P (2003) Interplay between syntax and semantics during sentence comprehension: ERP effects of combining syntactic and semantic violations. Journal of cognitive neuroscience 15: 883–899. 14511541 10.1162/089892903322370807

[2] Kuperberg GR (2007) Neural mechanisms of language comprehension: Challenges to syntax. Brain Research 1146: 23–49. 17400197 10.1016/j.brainres.2006.12.063

[3] Ye Z, Zhan W, Zhou X (2007) The semantic processing of syntactic structure in sentence comprehension: An ERP study. Brain research 1142: 135–145. 17303093 10.1016/j.brainres.2007.01.030

[4] Wang S, Zhu Z, Zhang JX, Wang Z, Xiao Z, et al (2008) Broca’s area plays a role in syntactic processing during Chinese reading comprehension. Neuropsychologia 46: 1371–1378. 10.1016/j.neuropsychologia.2007.12.020 18255103

[5] Yu J, Zhang Y (2008) When Chinese semantics meets failed syntax. NeuroReport 19: 745–749. 10.1097/WNR.0b013e3282fda21d 18418250

[6] Wang S, Mo D, Xiang M, Xu R, Chen H-C (2013) The time course of semantic and syntactic processing in reading Chinese: Evidence from ERPs. Language and Cognitive Processes: 1–20.

[7] Ferreira F, Clifton C Jr (1986) The independence of syntactic processing. Journal of memory and language 25: 348–368.

[8] Dapretto M, Bookheimer SY (1999) Form and content: dissociating syntax and semantics in sentence comprehension. Neuron 24: 427–432. 10571235 10.1016/s0896-6273(00)80855-7

[9] Osterhout L, Nicol J (1999) On the distinctiveness, independence, and time course of the brain responses to syntactic and semantic anomalies. Language and Cognitive Processes 14: 283–317.

[10] Braze D, Shankweiler D, Ni W, Palumbo LC (2002) Readers’ eye movements distinguish anomalies of form and content. Journal of Psycholinguistic Research 31: 25–44. 11924838 10.1023/a:1014324220455 PMC2850050

[11] Friederici AD, Rüschemeyer S-A, Hahne A, Fiebach CJ (2003) The role of left inferior frontal and superior temporal cortex in sentence comprehension: localizing syntactic and semantic processes. Cerebral cortex 13: 170–177. 12507948 10.1093/cercor/13.2.170

[12] Hahne A, Friederici AD (2002) Differential task effects on semantic and syntactic processes as revealed by ERPs. Cognitive Brain Research 13: 339–356. 11918999 10.1016/s0926-6410(01)00127-6

[13] Friederici A, Gunter T, Hahne A, Mauth K (2004) The relative timing of syntactic and semantic processes in sentence comprehension. NeuroReport 15: 165–169. 15106851 10.1097/00001756-200401190-00032

[14] Ye Z, Luo Y-j, Friederici AD, Zhou X (2006) Semantic and syntactic processing in Chinese sentence comprehension: Evidence from event-related potentials. Brain research 1071: 186–196. 16412999 10.1016/j.brainres.2005.11.085

[15] Friederici AD, Weissenborn J (2007) Mapping sentence form onto meaning: The syntax—semantic interface. Brain Research 1146: 50–58. 16956590 10.1016/j.brainres.2006.08.038

[16] Steinhauer K, Drury JE (2012) On the early left-anterior negativity (ELAN) in syntax studies. Brain and language 120: 135–162. 10.1016/j.bandl.2011.07.001 21924483

[17] Rayner K, Carlson M, Frazier L (1983) The interaction of syntax and semantics during sentence processing: Eye movements in the analysis of semantically biased sentences. Journal of verbal learning and verbal behavior 22: 358–374.

[18] Guntkr TC, Stowe LA, Mulder G (1997) When syntax meets semantics. Psychophysiology 34: 660–676. 9401421 10.1111/j.1469-8986.1997.tb02142.x

[19] Chen H-C, Zhou X (1999) Processing east Asian languages: An introduction. Language and Cognitive Processes 14: 425–428.

[20] Zhang Y, Zhang J (2008) Brain responses to agreement violations of Chinese grammatical aspect. NeuroReport 19: 1039–1043. 10.1097/WNR.0b013e328302f14f 18580575

[21] Yang J, Wang S, Chen H-C, Rayner K (2009) The time course of semantic and syntactic processing in Chinese sentence comprehension: Evidence from eye movements. Memory & cognition 37: 1164–1176. 10.1016/j.jecp.2014.12.001 19933459

[22] Zhou DX, Ye Z, Cheung H, Chen HC (2009) Processing the Chinese language: An introduction. Language and Cognitive Processes 24: 929–946.

[23] Zhang Y, Yu J, Boland JE (2010) Semantics does not need a processing license from syntax in reading Chinese. Journal of Experimental Psychology: Learning, Memory, and Cognition 36: 765 10.1037/a0019254 20438271

[24] Chen S, Bates E (1998) The dissociation between nouns and verbs in Broca’s and Wernicke’s aphasia: Findings from Chinese. Aphasiology 12: 5–36.

[25] Liu Y, Hua S, Weekes BS (2007) Differences in neural processing between nouns and verbs in Chinese: Evidence from EEG. Brain and Language 103: 75–77.

[26] Zhang Y, Li P, Piao Q, Liu Y, Huang Y, et al (2013) Syntax does not necessarily precede semantics in sentence processing: ERP evidence from Chinese. Brain and language 126: 8–19. 10.1016/j.bandl.2013.04.001 23648559

[27] Friederici AD, Frisch S (2000) Verb argument structure processing: The role of verb-specific and argument-specific information. Journal of Memory and Language 43: 476–507.

[28] Frisch S, Hahne A, Friederici AD (2004) Word category and verb—argument structure information in the dynamics of parsing. Cognition 91: 191–219. 15168895 10.1016/j.cognition.2003.09.009

[29] Rösler F, Pütz P, Friederici A, Hahne A (1993) Event-related brain potentials while encountering semantic and syntactic constraint violations. Cognitive Neuroscience, Journal of 5: 345–362. 10.1162/jocn.1993.5.3.345 23972222

[30] Tanenhaus MK, Boland J, Garnsey SM, Carlson GN (1989) Lexical structure in parsing long-distance dependencies. Journal of psycholinguistic research 18: 37–50. 2926695 10.1007/BF01069045

[31] Hagoort P, Brown C, Groothusen J (1993) The syntactic positive shift (SPS) as an ERP measure of syntactic processing. Language and cognitive processes 8: 439–483.

[32] Bornkessel-Schlesewsky I, Kretzschmar F, Tune S, Wang L, Genç S, et al (2011) Think globally: Cross-linguistic variation in electrophysiological activity during sentence comprehension. Brain and Language 117: 133–152. 10.1016/j.bandl.2010.09.010 20970843

[33] Ouyang G, Herzmann G, Zhou C, Sommer W (2011) Residue iteration decomposition (ride): a new method to separate erp components on the basis of latency variability in single trials. Psychophysiology 48: 1631–1647. 10.1111/j.1469-8986.2011.01269.x 21895682

[34] Ouyang G, Schacht A, Zhou C, Sommer W (2013) Overcoming limitations of the ERP method with Residue Iteration Decomposition (RIDE): A demonstration in go/no‐go experiments. Psychophysiology 50: 253–265. 10.1111/psyp.12004 23316862

[35] Stürmer B, Ouyang G, Zhou C, Boldt A, Sommer W (2013) Separating stimulus‐driven and response‐related LRP components with Residue Iteration Decomposition (RIDE). Psychophysiology 50: 70–73. 10.1111/j.1469-8986.2012.01479.x 23153305

[36] Verleger R, Metzner M, Ouyang G, Smigasiewicz K, Zhou C (2014) Testing the stimulus-to-response bridging function of the oddball-P3 by delayed response signals and residue iteration decomposition (RIDE). NeuroImage 100: 271–280. 10.1016/j.neuroimage.2014.06.036 24960419

[37] Proverbio AM, Riva F (2009) RP and N400 ERP components reflect semantic violations in visual processing of human actions. Neuroscience Letters 459: 142–146. 10.1016/j.neulet.2009.05.012 19427368

[38] Friederici AD (1995) The time course of syntactic activation during language processing: A model based on neuropsychological and neurophysiological data. Brain and language 50: 259–281. 7583190 10.1006/brln.1995.1048

[39] Sitnikova T, Holcomb PJ, Kiyonaga KA, Kuperberg GR (2008) Two neurocognitive mechanisms of semantic integration during the comprehension of visual real-world events. Journal of cognitive neuroscience 20: 2037–2057. 10.1162/jocn.2008.20143 18416681 PMC2673092

[40] Zhou X, Jiang X, Ye Z, Zhang Y, Lou K, et al (2010) Semantic integration processes at different levels of syntactic hierarchy during sentence comprehension: An ERP study. Neuropsychologia 48: 1551–1562. 10.1016/j.neuropsychologia.2010.02.001 20138898

[41] Osterhout L, Holcomb PJ (1992) Event-related brain potentials elicited by syntactic anomaly. Journal of Memory and Language 31: 785–806.

[42] Kaan E, Swaab TY (2003) Repair, revision, and complexity in syntactic analysis: An electrophysiological differentiation. Journal of Cognitive Neuroscience 15: 98–110. 12590846 10.1162/089892903321107855

[43] Kuperberg GR, Caplan D, Sitnikova T, Eddy M, Holcomb PJ (2006) Neural correlates of processing syntactic, semantic, and thematic relationships in sentences. Language and cognitive processes 21: 489–530.

[44] Fiebach CJ, Schlesewsky M, Friederici AD (2002) Separating syntactic memory costs and syntactic integration costs during parsing: The processing of German WH-questions. Journal of Memory and Language 47: 250–272.

[45] Kuperberg GR, Sitnikova T, Caplan D, Holcomb PJ (2003) Electrophysiological distinctions in processing conceptual relationships within simple sentences. Cognitive Brain Research 17: 117–129. 12763198 10.1016/s0926-6410(03)00086-7

[46] Kuperberg GR, Kreher DA, Sitnikova T, Caplan DN, Holcomb PJ (2007) The role of animacy and thematic relationships in processing active English sentences: Evidence from event-related potentials. Brain and language 100: 223–237. 16546247 10.1016/j.bandl.2005.12.006

[47] Chow W-Y, Phillips C (2013) No semantic illusions in the “Semantic P600” phenomenon: ERP evidence from Mandarin Chinese. brain research 1506: 76–93. 10.1016/j.brainres.2013.02.016 23422676

[48] Kolk HH, Chwilla DJ, van Herten M, Oor PJ (2003) Structure and limited capacity in verbal working memory: A study with event-related potentials. Brain and language 85: 1–36. 12681346 10.1016/s0093-934x(02)00548-5

